# Cohort comparison study of cardiac disease and atherosclerotic burden in type 2 diabetic adults using whole body cardiovascular magnetic resonance imaging

**DOI:** 10.1186/s12933-015-0284-2

**Published:** 2015-09-18

**Authors:** Suzanne L. Duce, Jonathan R. Weir-McCall, Stephen J. Gandy, Shona Z. Matthew, Deirdre B. Cassidy, Lynne McCormick, Petra Rauchhaus, Helen Looker, Helen M. Colhoun, J. Graeme Houston

**Affiliations:** Division of Cardiovascular and Diabetes Medicine, Medical Research Institute, University of Dundee, Level 7, Ninewells Hospital, Dundee, DD1 9SY UK; NHS Tayside Clinical Radiology, Ninewells Hospital, Dundee, DD1 9SY UK; NHS Tayside Medical Physics, Ninewells Hospital, Dundee, DD1 9SY UK; Dundee Epidemiological and Biostatistics Unit, University of Dundee, Dundee, DD1 9SY UK; Division of Population Health Sciences, Medical Research Institute, University of Dundee, The Mackenzie Building, Dundee, DD2 4BF UK

**Keywords:** Whole body MRI, Magnetic resonance angiography, CMR, LVA, WB CVMR, Cardiovascular disease, Atherosclerosis, Atheroma score, Type 2 diabetes mellitus, T2DM

## Abstract

**Background:**

Whole body cardiovascular MR (WB CVMR) combines whole body angiography and cardiac MR assessment. It is accepted that there is a high disease burden in patients with diabetes, however the quantification of the whole body atheroma burden in both arterial and cardiac disease has not been previously reported. In this study we compare the quantified atheroma burden in those individuals with and without diabetes by clinical cardiovascular disease (CVD) status.

**Methods:**

158 participants underwent WB CVMR, and were categorised into one of four groups: (1) type 2 diabetes mellitus (T2DM) with CVD; (2) T2DM without CVD; (3) CVD without T2DM; (4) healthy controls. The arterial tree was subdivided into 31 segments and each scored according to the degree of stenosis. From this a standardised atheroma score (SAS) was calculated. Cardiac MR and late gadolinium enhancement images of the left ventricle were obtained for assessment of mass, volume and myocardial scar assessment.

**Results:**

148 participants completed the study protocol—61 % male, with mean age of 64 ± 8.2 years. SAS was highest in those with cardiovascular disease without diabetes [10.1 (0–39.5)], followed by those with T2DM and CVD [4 (0–41.1)], then those with T2DM only [3.23 (0–19.4)] with healthy controls having the lowest atheroma score [2.4 (0–19.4)]. Both groups with a prior history of CVD had a higher SAS and left ventricular mass than those without (p < 0.001 for both). However after accounting for known cardiovascular risk factors, only the SAS in the group with CVD without T2DM remained significantly elevated. 6 % of the T2DM group had evidence of silent myocardial infarct, with this subcohort having a higher SAS than the remainder of the T2DM group [7.7 (4–19) vs. 2.8 (0–17), p = 0.024].

**Conclusions:**

Global atheroma burden was significantly higher in those with known cardiovascular disease and without diabetes but not in those with diabetes and cardiovascular disease suggesting that cardiovascular events may occur at a lower atheroma burden in diabetes.

**Electronic supplementary material:**

The online version of this article (doi:10.1186/s12933-015-0284-2) contains supplementary material, which is available to authorized users.

## Background

Type 2 diabetes mellitus (T2DM) is a chronic metabolic disease resulting in an inability to control glucose homeostasis due to insulin resistance. It accounts for over 90 % of all diabetes cases [1], and in many western countries it is reaching epidemic proportions due to an ageing population, increasing prevalence of obesity and sedentary lifestyles [2]. There are several clinical complications associated with diabetes which include macro- and micro-vascular disorders such as stroke, coronary heart disease, peripheral vascular disease as well as nephropathy and retinopathy [3]. These conditions can impose an immense burden on the quality of life of individuals with diabetes [4].

The systemic nature of atherosclerosis is widely appreciated with disease in one arterial territory predictive of disease in another, with this effect particularly pronounced in diabetes [5]. Thus the ability to assess the whole body burden of this disease process is desirable in an effort to greater understand macro-vascular disease burden and behaviour in diabetes, as well as to help quantify future risk, target treatments and monitor response. Whole body cardiovascular magnetic resonance imaging (WB CVMR) combines cardiac magnetic resonance (CMR) and whole body MR angiography (WB-MRA) sequences to produce high spatial and temporal resolution images of the cardiovascular system with coverage from the head to the feet [6]. It has been shown to be effective at systemically quantifying the presence and severity of cardiac disease and atherosclerosis across the arterial network in patients with cardiovascular disease in single examination taking of the order of 45 min [7]. WB-MRA in a diabetic population has been previously demonstrated to be a feasible single examination for ascertaining cardiovascular disease distribution, detecting occult disease and quantifying stenotic burden, and for these findings to provide strong prognostic information of future cardiovascular events [8, 9].

While previous studies have documented the burden of vascular disease in people with diabetes, to date no study has focussed on the impact of type 2 diabetes on the whole body atheroma burden combining quantitative of both WB-MRA and CMR. Thus we aimed to compare the effects of type 2 diabetes and individuals without CVD on the quantitative atherosclerotic burden throughout the body in those with and without known cardiovascular disease.

## Methods

### Ethical approval

The East of Scotland Research Ethics Committee approved the protocols. The study was conducted at in accordance with the Declaration of Helsinki. The volunteers gave written informed consent to participate in this study.

### Participants

The study was a single centre observational sub-study of the multicentre SUrrogate markers for Micro- and Macrovascular hard endpoints for Innovative diabetes Tools (SUMMIT) study. 158 volunteers were recruited, with 148 completing the WB-CVMR protocol between March 2009 and December 2012.

The participants were recruited from the UK Type 2 Diabetes Case–Control Collection Wellcome study and via the Scottish Primary Care Research network, the Scottish Diabetes Research network, Secondary Care Diabetes clinics, and through advertising such as posters and leaflets. After clinical evaluation, the subjects were categorised into four groups based on their history of type 2 diabetes and cardiovascular disease as follows: *Group 1* type 2 diabetes mellitus (T2DM) with a prior clinical diagnosis of cardiovascular disease that included coronary artery disease (CAD), cerebrovascular disease and/or lower extremity arterial disease (LEAD); *Group 2* type 2 diabetes mellitus with no clinical evidence of cardiovascular disease; *Group 3* absence of diabetes mellitus with clinical evidence of CAD, cerebrovascular disease and/or LEAD; *Group 4* healthy controls, with no biochemical evidence of diabetes mellitus (see below) and no clinical evidence of cardiovascular disease.

The inclusion criteria for Groups 1 and 2 (with T2DM) was T2DM diagnosis after the age of 35 and no requirement for insulin injection for 12 months after diagnosis. The non-diabetic status of those in Groups 3 and 4 were confirmed by testing their haemoglobin A1C (HbA1C) levels, with inclusion criteria requiring levels of <6.5 % (48 mmol/ml). Coronary arterial disease inclusion criteria were non-fatal acute myocardial infarction, hospitalised acute coronary syndrome, resuscitated cardiac arrest (not attributed to non CAD causes), coronary artery bypass graft (CABG) or any other coronary revascularisation procedure. Cerebrovascular disease inclusion criteria were non-fatal strokes and transient ischemic attacks (TIA) confirmed by a specialist stroke physician. It excluded TIA not confirmed by specialist, haemorrhagic stroke, and stroke associated with a primary haematological disease e.g. leukaemia, polycythaemia, blood disease, tumours, trauma or surgical procedures. Lower extremity arterial disease (LEAD) inclusion criteria were clinical intermittent claudication with either ankle-brachial pressure index (ABPI) <0.9, abnormal toe systolic pressure, pulse volume recordings, transcutaneous oxygen measurements, vascular imaging, or prior corrective surgery, angioplasty or above ankle amputation (see Additional file 1: Table S1 for tabulated inclusion criteria for the cardiovascular disease groups). Exclusion criteria included the presence of metallic implants, history of claustrophobia, pregnancy, renal replacement or eGFR <30 ml/min/1.73 m^2^, and therapy for any chronic inflammatory disease, atrial fibrillation or malignancy.

### Magnetic resonance imaging

Images were acquired on a 32 RF receiver channel, 3 Tesla MRI scanner (Magnetom Trio, Siemens, Erlangen, Germany). For whole body coverage, a combination of six RF coils including head matrix, neck matrix, spine matrix, two body matrix and peripheral angiography phased array RF surface coils were used. Subjects where placed supine, head first into the magnet bore. The imaging protocol was carried out in three phases: (1) MRA of the thoracic and neck, and distal lower limbs, (2) CMR including late gadolinium enhancement (LGE) and (3) MRA of the abdomen, pelvis and proximal lower limb.

#### Whole body magnetic resonance angiography protocol

Whole body magnetic resonance angiography (WB-MRA) images involved the acquisition of four overlapping 3D data sets using a coronal spoiled FLASH sequence (repetition time TR = 2.6–3.47 ms; echo time TE = 0.96–1.21 ms; flip angle = 16°–37°; pixel area = 1.1–1.5 mm^2^ and slice thickness = 1–1.4 mm, slight variation according to station and participant body habitus). Anatomical paired images were acquired pre- and post-contrast from four anatomically distinct stations—head, neck and thorax (station 1), abdomen and pelvis (station 2), upper legs (stations 3) and lower legs (station 4). 0.5 M gadoterate meglumine (Guerbet, Villepinte, France) was administered by intravenous injection into antecubital fossa using a Spectris Solaris power injector (MedRad, Pittsburgh, USA) at a rate of 1.5 ml/s, followed by a 20 ml bolus of saline. A dual injection, two stage acquisition with uptitring of contrast volume was performed due to the benefits of this technique on image quality over a single injection technique [10, 11]. Stations 1 and 4 pre-contrast images were acquired following which post-contrast images were acquired after an injection of 10 ml gadoterate meglumine with a 20 ml saline flush, both administered at 1.5 ml/s. Cardiac MRI was performed after this first injection before stations 2 and 3 image acquisition began. Pre contrast images were acquired of both stations following which post-contrast images were acquired after an injection of 15 ml gadoterate meglumine and 20 ml saline flush administered at 1.5 ml/s.

#### Cardiac magnetic resonance (CMR) protocols

Cardiac magnetic resonance (CMR) imaging utilised a spine matrix and six-element body array matrix RF coils. TurboFLASH two-chamber, four-chamber and short axis localiser, and two-chamber and four-chamber cine images were acquired. Left ventricular assessment involved the acquisition of a horizontal long axis, vertical long axis and stacked short axis cine images with repeated end-expiratory breath-holds, from the atrio-ventricular ring to the apex. These were acquired using a CINE TrueFISP sequence with retrospective ECG-gating (repetition time TR = 3.4 ms; echo time TE = 1.48 ms; flip angle = 50°–60°; pixel area = 1.4 mm × 1.9 mm; slice thickness = 6 mm; inter-slice gap = 4 mm). Ten minutes after the injection of the first dose of contrast agent the LGE images were acquired using a 2D phase sensitive inversion recovery (PSIR) sequence (repetition time TR = 846.4/5.21 ms; echo time TE = 1.99 ms; flip angle = 20^0^; pixel area = 1.4 mm × 1.9 mm; slice thickness = 6 mm; inter-slice gap = 4 mm). The mean inversion time (TI) was 376 ms (range 300–450 ms).

#### WB-MRA image analysis

**Fig. 1:**
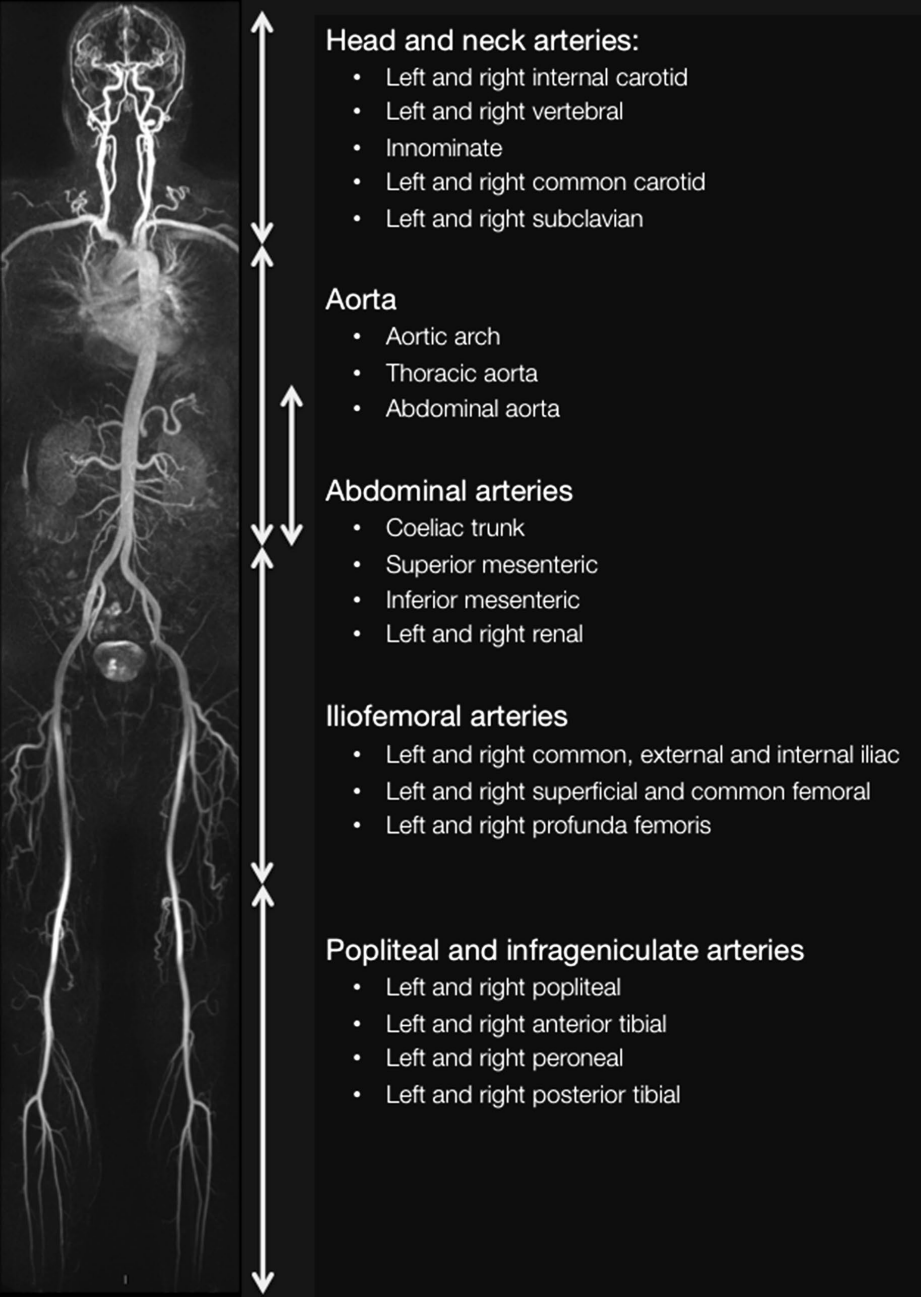
Whole body angiogram with stations and vascular territories described

**Fig. 2:**
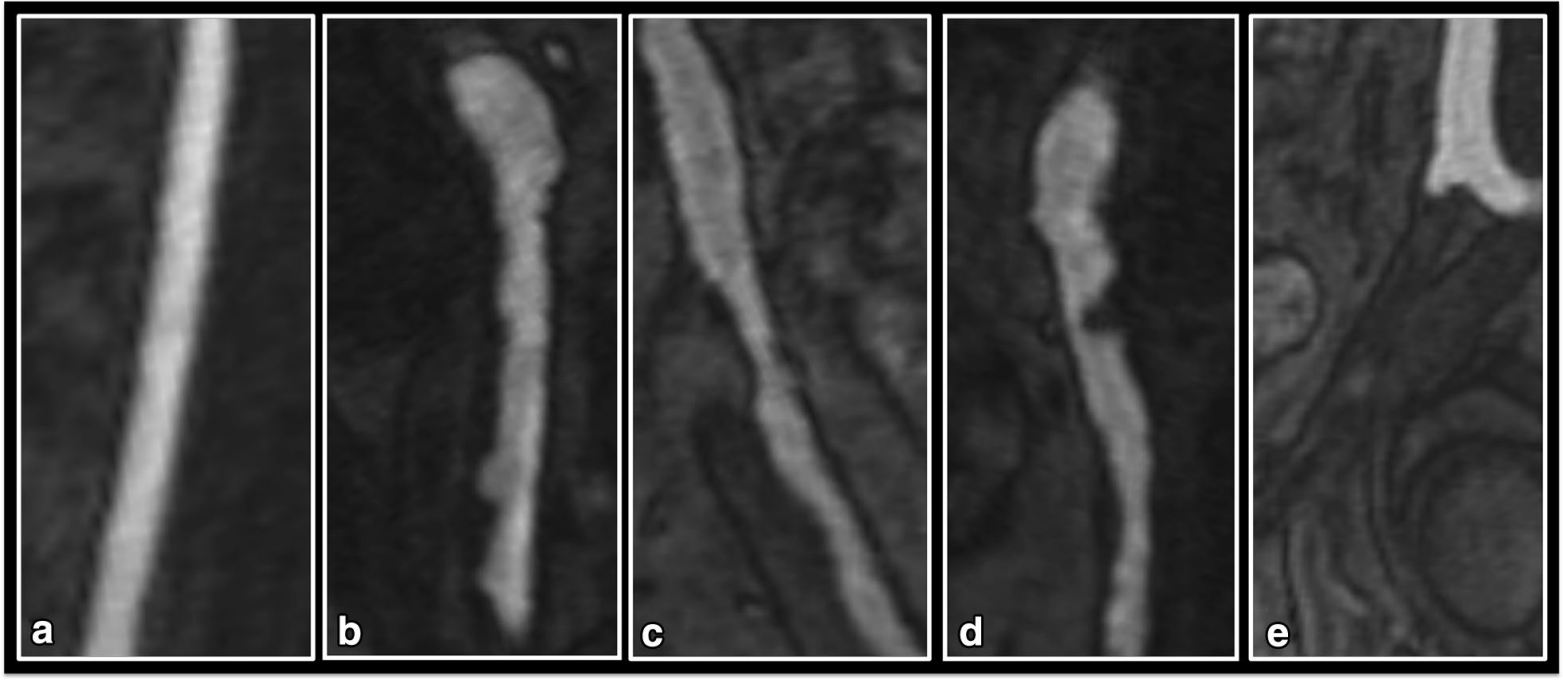
MRA visualization of the iliac arteries demonstrating examples of each of the five categorical vessel scores. **a** Grade 0. **b** Grade 1—diffuse irregularity of the vessel wall, none of which is causing >50 % stenosis. **c** Grade 2—50–70 % stenosis in the proximal external iliac. **d** Grade 3—>70 % stenosis in the common iliac. **e** Grade 4—complete occlusion of the external iliac just after the bifurcation of the common iliac with compensatory dilation of the internal iliac artery evident

The 3D WB-MRA datasets were viewed offline (Carestream PACS Client Suite Version 10.1 sp1, Rochester, NY, USA) as source images using both multi-planar reconstruction (MPR) and maximum intensity projections (MIP) by a radiologist (JRWM) with experience of reporting over 400 whole body magnetic resonance angiograms. The radiologist was blinded to the participants’ clinical history during image analysis. The arterial network was divided into 31 vessel segments extending from the distal internal carotid arteries to the distal points of the calf vessels (Fig. 1). Each arterial segment was visually inspected and assessed as either ‘diagnostic’ or ‘non-diagnostic’ depending on image quality and the clarity of vascular structure. Non-diagnostic segments were assigned as non-interpretable. The diagnostic segments were scored according to the degree of narrowing of the lumen diameter, with stenosis graded at the narrowest part of the vessel. Where more than one stenosis was present in any vessel, the most severe stenosis was used to score the vessel. Categorical scores from 0 to 4 were allocated to each vessel segment, where 0, segment with no stenosis; 1, <50 % stenosis; 2, 51–70 % stenosis; 3, 71–99 % stenosis; 4, vessel occlusion (Fig. 2). The ‘standardised atheroma score’ (SAS) was calculated by summing each individual segment’s stenosis score, and divided by the number of diagnostic segments (n) before dividing by 4 which is the maximum potential score (Eq. 1): [7, 12].

1 $$SAS = \left[ {\left( {\frac{\Sigma \, MRA \,score}{n} } \right) \div 4} \right] \times 100$$

The 31 vessel segments were subdivided into five anatomical territories: (1) the head and neck arteries, (2) the aorta, (3) the abdominal arteries, (4) the ilio-femoral arteries and (5) Popliteal and infrageniculate arteries (Fig. 1). Regional SASs were calculated for each anatomical territory.

20 datasets were chosen at random and rescored by the same observer to derive intra-observer agreement.

#### CMR image analysis

Left ventricular analysis images were analysed offline using Argus software (Siemens, Erlangen, Germany) by an experienced researcher (SZM). Segmentation involved tracing endocardial and epicardial contours on the short-axis left ventricle images at end-diastolic and end-systolic phases of the cardiac cycle. Papillary muscles were treated as part of the blood pool volume unless they were indistinguishable from the myocardial wall, and then they were assigned as left ventricle muscle. The left ventricular mass (LVM), stroke volume (LVSV), ejection fraction (LVEF), end-diastolic (LVEDV) and end-systolic volumes (LVESV) were determined using algorithm based on the Simpson rule. Results were normalised to body surface area using the DuBois formula [13]. Left ventricular mass volume ratio (LVMVR) was calculated as LVEDV/LVM. Left ventricular global function index (LVGFI) was calculated as LVGFI = (LVSV/LV global volume) × 100, where LV global volume was defined as the sum of the LV mean cavity volume [(LVEDV + LVESV)/2] and the myocardium volume [14]. The PSIR sequences of the left ventricle were inspected for evidence of late gadolinium enhancement (LGE) using an imaging workstation (Carestream, Rochester, NY, USA). 10 datasets were chosen at random and rescored by the same observer to derive intra-observer agreement.

### Statistical methods

Descriptive statistics were used for the analysis of the demographic and clinical features of the cohorts with data expressed as mean ± standard deviation (SD) for normally distributed data, and median (interquartile range) for non-normal distributed data. Normality tests were performed; if the test failed, where possible standard transformations such as square root, reciprocal or logarithmic transforms were used to generate a Gaussian distribution. To test the null hypothesis to determine if samples originate from the same distribution, the one-way analysis of variance (ANOVA) with the Bonferroni post hoc adjustment was used for the parametric data and Kruskal–Wallis ANOVA by ranks was used for the non-parametric data. ANCOVA was performed to confirm differences between the groups with the WB-SAS and LVM used separately as the dependant variables correcting for age, sex, BMI, history of hypertension and smoking status. Two-way random effect absolute agreement single measure intraclass correlation co-efficient (ICC) was performed to assess intra-observer consistency of the WB-SAS and LV metrics. All data were analysed using SPSS statistical package (version 21.0, SPSS Inc. Chicago, IL, USA). Significance was assumed when p < 0.05. A statistician from the Dundee Epidemiology and Biostatistics Unit (PR) provided statistical support.

## Results

Of the 148 participants who completed the study protocol, 87 (61 %) were male with a mean age was 64 ± 8.2 years (see Table 1 for full demographic details). The group with cardiovascular disease and diabetes had a significantly greater proportion of males, while the healthy volunteers had a significantly lower BMI and prevalence of hypertension compared to the other three groups. Other than these there were no statistically significant differences in the demographic metrics between each of the groups. In particular there was no difference in duration of diabetes, or in HbA1c between the diabetic groups with and without history of cardiovascular disease.

**Table 1:** Population demographics of the participants

	Group 1 CVD+ DM+	Group 2 CVD− DM+	Group 3 CVD+ DM−	Group 4 CVD− DM−	p value
N	34	55	30	29	
Male (%)	76*	56	73	41	0.015
Age (years)	65 ± 7	63 ± 8	67 ± 9**	62 ± 8	0.03
BMI (kg/m^2^)	31 ± 4*	30 ± 6*	29 ± 4	28 ± 4	0.009
Hypertension	24 (75 %)*	34 (62 %)^**^**^	27 (90 %)*	7 (24 %)	<0.001
Systolic BP	132 ± 13	137 ± 14	137 ± 15	133 ± 15	0.4
Diastolic BP	74 ± 8	78 ± 8	76 ± 9	78 ± 9	0.25
LDL-cholesterol (mmol/l)	1.69 ± 0.5*	1.98 ± 0.8*	1.97 ± 0.6*	2.80 ± 0.8	<0.001
HDL-cholesterol (mmol/l)	1.11 ± 0.3*	1.26 ± 0.3*	1.27 ± 0.5	1.53 ± 0.4	0.001
Triglycerides (mmol/l)	2.08 ± 1.0	1.70 ± 0.8	1.60 ± 0.9	1.76 ± 1.0	0.18
Creatinine	86.3 ± 24	73.1 ± 17	80.7 ± 17	70.6 ± 15	0.003
HbA1c	7.3 (6–11)**^**	7.3 (5–12)**^**	5.7 (5–8)	5.6 (5–6)	<0.001
Duration of diabetes (years)	10.4 ± 4.7	9 ± 6.2			0.3
Current/ex smoker (%)	72	49	60	52	0.37
Medications					
Antihypertensive therapy (%)	84**	56*	93**	28	<0.001
Number antihypertensive agents	1.83 ± 1.0**	0.96 ± 1.0	1.93 ± 0.9**	0.39 ± 0.1	<0.001
Statin	88*	73*	80*	25	<0.001
Prior cardiovascular events^a^					
CAD	75		77		0.88
Cerebrovascular	19		23		0.66
LEAD	19		17		0.83

Values expressed as mean ± SD, or N (%)

Group 1, T2DM and CVD; Group 2, T2DM, no CVD; Group 3, CVD; Group 4, healthy volunteers

*BMI* body mass index, *BP* blood pressure, *CAD* coronary artery disease, *LEAD* lower extremity arterial disease

* p < 0.05 compared to Group 4

** p < 0.05 compared to Groups 2 and 4

^^^p < 0.05 compared to Groups 3 and 4

^a^Groups add up to >100 % as several individuals had more than one prior cardiovascular event

On WB-MRA analysis, 4564 of the 4588 vessel segments (31 segments in 143 patients) evaluated were graded as ‘diagnostic’ quality, giving a diagnostic rate of 99.5 %. 24 segments in six examinations were rated as ‘non-diagnostic’ due to movement artefact or incomplete vessel visualisation. 961 (21.1 %) of the 4564 WB-MRA diagnostic segments had evidence of luminal narrowing: 769 (10.3 %) had stenosis below 50 %, 75 (1.6 %) had stenosis between 50 and 70 %, 60 (1.3 %) had stenosis between 70 and 99 %, and 57 (1.2 %) had complete occlusion. 135 participants (91 %) had evidence of at least one stenotic vessel (Table 2).

**Table 2:** Comparison of MRI metrics between the four groups

	Group 1 CVD+ DM+	Group 2 CVD− DM+	Group 3 CVD+ DM−		Group 4 CVD− DM−	*p* value
WB-MRA						
WB SAS	4 (0–41.1)**	3.23 (0–19.4)	10.1 (0–39.5)**		2.4 (0–19.4)	<0.001
Head/neck-SAS	5.6 (0–11.8)**^**	2.8 (2.8–5.6)	5.6 (2.8–9.0)		0 (0–5.6)	0.024
Aorta-SAS	8.3 (0–16.7)	0 (0–8.3)	16.7 (8.3–16.7)**		8.3 (0–8.3)	<0.001
Abdomen-SAS	0 (0–10)	0 (0–5)	5 (0–15)*		0 (0–10)	0.02
Ilio-femoral-SAS	4.2 (0–22.9)*	4.2 (0–8.3)	18.8 (4.2–30.2)**		0 (0-10.4)	<0.001
Run off-SAS	4.7 (0–19.5)**	0 (0–3.1)	3.1 (0–25.8)**		0 (0–1.6)	<0.001
Normal vessels	765**	1432	627**		779	<0.001
Vessels with 1–50 % stenosis	190	251	221**		107	0.008
Vessels with 51–70 % stenosis	38**	7	24*		6	<0.001
Vessels with 71–99 % stenosis	24*	4	26**		6	0.002
Occluded vessels	22	4	30**		1	<0.001
Abnormal vessels	8.5**	5	10.1**		4.1	<0.001
N (%) with >50 % stenosis in any vessel	19 (56 %)**	12 (21.8 %)	20 (66.7 %)**		11 (38 %)	<0.001
Cardiac MRI						
LVM (g/m^2^)	60.5 (45–86)**	54.4 (39–95)	60 (41–86)**	50.9 (35–73)		*0.001*
LVEDV (ml/m^2^)	71.2 ± 14.5	66.9 ± 12.8	74.9 ± 19.8	68.7 ± 9.9		0.096
LVESV (ml/m^2^)	24.4 (9–58)	22.4 (11–55)	25.1 (11–90)	24.9 (13–38)		0.52
LVEF (%)	64.6 ± 11.3	65.9 ± 8.8	64.3 ± 10.3	66 ± 7.4		0.83
LVSV (ml/m^2^)	45.3 ± 9.2	43.7 ± 8.2	47 ± 8.2	45.1 ± 7.2		0.37
LVMVR (g/ml)	0.88 ± 0.14**^**	0.84 ± 0.13	0.83 ± 0.16	0.78 ± 0.14		0.036
LVGFI	0.43 ± 0.09	0.45 ± 0.08	0.44 ± 0.08	0.47 ± 0.07		0.21
LGE (%)	9 (29)*	3 (5.5)	11 (38)*	0 (0)		<0.001

Values expressed as mean ± SD, median (range) or N (%)

Group 1, T2DM and CVD; Group 2, T2DM, no CVD; Group 3, CVD; Group 4, healthy volunteers

*SAS* standardised atheroma score, *WB* whole body, *LVM* left ventricular mass^a^, *EDV* end diastolic volume^a^, *ESV* end systolic volume^a^, *EF* ejection fraction, *SV* stroke volume^a^, *LVMVR* left ventricular mass volume ratio, *LVGFI* left ventricular global function index, *LGE* late gadolinium enhancement

* p < 0.05 when compared to Group 2

** p < 0.05 when compared to Groups 2 and 4

^**^**^p < 0.05 when compared to Group 4

^a^Normalised to body surface area

**Fig. 3:**
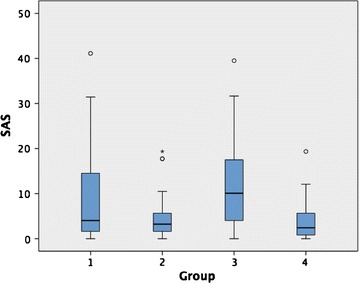
*Box plot* comparing SAS between the four groups. Group 1, T2DM and CVD; Group 2, T2DM, no CVD; Group 3, CVD; Group 4, healthy volunteers. *Box* 1st–3rd quartiles with *thick line* representing the group median. *Open circle* and *asterisk* outliers

Those with cardiovascular disease without diabetes had the highest whole body standardised atheroma score (WB-SAS) of 10.1 (0–39.5) followed by those with cardiovascular disease and diabetes with a WB-SAS of 4 (0–41.1). The normal healthy volunteer group had the lowest WB-SAS of 2.4 (0–19.4), which was slightly lower than those with diabetes but no cardiovascular disease. The WB-SAS of the two cardiovascular groups (Groups 1, 3) was significantly higher than those of the healthy diabetic and healthy control groups (p ≤ 0.05) (Fig. 3). On univariate analysis of covariance after accounting for age, sex, BMI, history of hypertension and smoking status, the only significant difference that persisted was between the non-diabetic cardiovascular disease group and the healthy controls. There were no significant differences in WB-SAS between the cardiovascular disease groups with and without diabetes, nor between the diabetics without cardiovascular disease and the healthy controls.

Indexed left ventricular mass (LVM) and prevalence of late gadolinium enhancement was significantly higher in the two cardiovascular disease cohorts (p < 0.05) compared to both the non-cardiovascular disease groups (see Table 2). Left ventricular mass volume ratio (LVMVR) was significantly higher in the cardiovascular disease group with diabetes compared with the healthy controls but not compared to either of the other two groups. Difference between groups for LVM and LVMVR were lost after accounting for age, sex, BMI, history of hypertension and smoking status on analysis of covariance. There was no significant difference in left ventricular end diastolic volume (EDV) end systolic volume (ESV), stroke volume (SV), ejection fraction (EF) or left ventricular global function index (LVGFI) between the 4 groups. There were no significant differences in any of the LV metrics between subjects with diabetes and those without when history of cardiovascular disease was accounted for.

Left ventricular myocardial contrast enhancement was observed in 29 % of type 2 diabetic with CVD (Group 1), 5.5 % of type 2 diabetic without CVD (Group 2), 39 % of non-diabetics with CVD (Group 3), and 0 % of non-diabetics with no CVD (Group 4) (Table 2). Clinical records showed that all 11 non-diabetic Group 3 subjects with positive LGE had a history of coronary heart disease. However only eight of the nine diabetic Group 1 subjects had a history of coronary heart disease, with one reporting known LEAD but no previous history of coronary artery disease in keeping with a silent myocardial infarct. 3 (5.5 %) participants with diabetes without any history of cardiac event demonstrated myocardial scarring typical of a myocardial infarction, the whole body SAS of these three individuals was significantly higher than the remainder of the diabetes group without history of cardiovascular disease [7.7 (4–19) vs. 2.8 (0–17), p = 0.024].

Both SAS and the LV metrics demonstrated good agreement on intra-observer analysis: SAS ICC 0.93 (95 % CI 0.83–0.97, p < 0.001); EF ICC 0.98 (95 % CI 0.85–0.99, p < 0.001); EDV ICC 0.98 (95 % CI 0.56–0.99, p < 0.001), SV ICC 0.98 (95 % CI 0.92–0.99, p < 0.001); LVM ICC 0.99 (95 % CI 0.98–0.99, p < 0.001). This is consistent with previous work where intra- and interobserver variability for both readers and techniques has been described in more detail [7].

Intra-observer variability was good for the WB-SAS (0.93 (95 % CI 0.83–0.97, p < 0.001).

## Discussion

In this study we have shown that in non-diabetics the global atheroma burden as measured by whole body MRA is significantly higher in individuals with clinically evident cardiovascular disease compared with diabetic and non-diabetic controls. However when looking at those with type 2 diabetes there was significant overlap in the atheroma burden between the groups with and without cardiovascular disease, and no significant difference between either groups compared with healthy controls. This is a surprising and, given the known effects of diabetic status on cardiovascular events, a slightly counterintuitive finding.

From the current observations it appears that either cardiovascular events occur at a lower atheroma burden in those with type 2 diabetes compared with non-diabetics, or that there is a clinical bias in the suspicion of vascular events in those with type 2 diabetes resulting in diagnosis of cardiovascular events at a lower disease threshold. Atherosclerotic plaques in those with diabetes have a larger necrotic core, a thinner capsule and impaired vascular repair mechanisms—all of which increase the likelihood of rupture [15–17]. Given that the majority of coronary and cerebrovascular events occur secondary to ruptured atherosclerotic plaque [18, 19], it is therefore feasible that cardiovascular events occur at a lower atheroma burden in a diabetic population due to a greater propensity of the plaques they do have of rupturing and occluding arteries. In addition diabetes is associated with impaired microcirculatory function, which itself is associated with a higher risk for future cardiovascular events, therefore potentially reducing the atheroma burden necessary to elicit a cardiovascular event [20, 21].

A previous study using whole body atheroma burden by Weckbach et al. [22] demonstrated an increased burden of vascular disease in a diabetic cohort compared with a healthy population. However given that the diabetic group included patients with cardiovascular disease while their control group excluded patients with cardiovascular disease it is difficult to extricate the effects of diabetes per se on atheroma burden, especially given that in our study we found a higher burden of atheroma in those with cardiovascular disease compared to those without. A separate subgroup analysis by the same group showed that those with diabetes with metabolic syndrome had a significantly higher atheroma burden than those without, but did not compare those with diabetes but without metabolic syndrome to the healthy cohort [8]. In a population based study of 306 70-year-old Hansen et al. found no association between diagnosis of diabetes and atheroma burden, however found a relationship between the HOMA insulin resistance index and atheroma score [23]. Lehrke et al. [24] also found no difference in atheroma burden between diabetic and non-diabetic groups, although the study only had 13 diabetic patients in their cohort. Consistent with these previous studies we have found that when diabetic patients are compared with patients without diabetes after stratifying them based on cardiovascular history that no difference in atheroma burden was observed. A similar lack of difference in stenotic atheroma burden has been observed on invasive coronary angiography between groups with and without type 2 diabetes [25, 26].

While our criteria for recruitment into the cardiovascular disease groups was strict it does not preclude the effects of referral bias in the type 2 diabetes cohort. Given the known increased cardiovascular risk in type 2 diabetes it is possible that the threshold for eliciting a referral for cardiovascular related investigations for symptomatology occurs at an earlier stage in those with diabetes than those without thus resulting in a lower observed atheroma burden. Greater prescription of antihypertensive and statin therapy was observed in both the groups with diabetes compared with the healthy controls, which if initiated early in the disease processes could also have attenuated disease progression given the known effects of statins on plaque volume [27].

Less likely is that the lack of difference in observed atheroma burden between the groups may lie in the lumenographic assessment that WB-MRA provides, with lack of ability to quantify non stenotic atheroma with positive remodelling. When considering coronary artery disease, catheter angiography has demonstrated no significant difference between diabetic and non-diabetic cohorts in stenotic disease burden [25, 26], however when intravascular ultrasound is used a greater plaque area encasing diffusely narrower arteries is observed [28]. Also CT coronary artery calcium scoring and CT coronary angiography, which can visualise extraluminal plaque, demonstrate higher plaque burden in diabetic cohorts [29]. The lumenographic nature of the technique is an acknowledged limitation [30], however no other widely available technique is yet available to quantify this metric throughout the entire body. Furthermore, atherosclerosis with positive vascular remodelling is a relatively early phenomenon in the development of plaque, therefore even if this were to be missed it would not explain the discrepancy in stenotic plaque burden.

Within the healthy diabetic cohort we observed a 5 % prevalence of silent myocardial infarct. This is in keeping with prior studies showing an incidence of silent infarcts in diabetic populations to range from 2.7 to 17 % depending on the method used and cohort studied [31–33]. Those with silent myocardial infarcts had a significantly higher atheroma burden which is consistent with other groups results showing that those with an elevated atheroma burden were more likely to have obstructive coronary artery disease and develop major adverse cardiovascular events at follow-up [9, 24]. This finding adds further support of the prognostic importance of the whole body atheroma burden on future cardiovascular events as evidenced by two prior studies showing the stenotic atheroma burden on WB-MRA to have superior prognostic efficacy over traditional risk scores, carotid intima media thickness and ankle-brachial pressure index [9, 34].

There are several limitations of the current study. As previously discussed whole body MRA is a lumenographic technique, and therefore may miss plaque with vascular remodelling which maintains the luminal diameter. Our scoring system prescribes no weighting to specific vessels, or to vessels with multisite stenosis. Long-term studies are required to assess the prognostic significance of disease of individual vessels, and how multiple lesions compare with single lesions in these different groups. Finally the coronary vessels are not assessed in the current study protocol. MRI coronary angiography holds some promise but is still some way off routine clinical use [35], however as this continues to improve incorporation of this in WB-CVMR will become an interesting avenue for exploration in the future.

## Conclusion

Global atheroma burden was significantly higher in those with known cardiovascular disease and without diabetes but not in those with diabetes and cardiovascular disease suggesting that cardiovascular events may occur at a lower atheroma burden in diabetes.

## Additional file

**Additional file 1: Table S1.** Breakdown of the inclusion criteria for each of the cardiovascular disease territories.

## Abbreviations

AHA: American Heart Association
CAD: coronary artery disease
CMR: cardiac magnetic resonance imaging
CVD: cardiovascular disease
LEAD: lower extremity arterial disease
LGE: late gadolinium enhancement
LVM: left ventricular mass
LVSV: left ventricular stroke volume
LVEF: left ventricular ejection fraction
LVEDV: left ventricular end-diastolic volume
LVESV: left ventricular end-systolic volume
LVMVR: left ventricular mass volume ratio
LVGFI: left ventricular global function index (LVGFI)
MRA: magnetic resonance angiography
PAD: peripheral arterial disease
SAS: standardised atheroma score
WB-CVMR: whole body magnetic cardiovascular magnetic resonance
WB-MRA: whole body magnetic resonance angiography

## References

[1] Alberti KG, Zimmet PZ (1998). Definition, diagnosis and classification of diabetes mellitus and its complications. Part 1: diagnosis and classification of diabetes mellitus provisional report of a WHO consultation. Diabet Med.

[2] Zimmet P, Alberti KG, Shaw J (2001). Global and societal implications of the diabetes epidemic. Nature.

[3] Danaei G, Lawes CMM, Vander Hoorn S, Murray CJL, Ezzati M (2006). Global and regional mortality from ischaemic heart disease and stroke attributable to higher-than-optimum blood glucose concentration: comparative risk assessment. Lancet.

[4] Rubin RR, Peyrot M (1999). Quality of life and diabetes. Diabetes Metab Res Rev..

[5] Bhatt DL, Eagle KA, Ohman EM, Hirsch AT, Goto S, Mahoney EM, Wilson PWF, Alberts MJ, D’Agostino R, Liau C-S, Mas J-L, Röther J, Smith SC, Salette G, Contant CF, Massaro JM, Steg PG (2010). Comparative determinants of 4-year cardiovascular event rates in stable outpatients at risk of or with atherothrombosis. JAMA.

[6] Ladd SC, Debatin JF, Stang A, Bromen K, Moebus S, Nuefer M, Gizewski E, Wanke I, Doerfler A, Ladd ME, Benemann J, Erbel R, Forsting M, Schmermund A, Jöckel K-H (2007). Whole-body MR vascular screening detects unsuspected concomitant vascular disease in coronary heart disease patients. Eur Radiol.

[7] Gandy SJ, Lambert M, Belch JJF, Cavin ID, Crowe E, Littleford R, Macfarlane JA, Matthew SZ, Martin P, Nicholas RS, Struthers AD, Sullivan F, Waugh SA, White RD, Weir-McCall JR, Houston JG (2015). Technical assessment of whole body angiography and cardiac function within a single MRI examination. Clin Radiol..

[8] Findeisen HM, Weckbach S, Stark RG, Reiser MF, Schoenberg SO, Parhofer KG (2010). Metabolic syndrome predicts vascular changes in whole body magnetic resonance imaging in patients with long standing diabetes mellitus. Cardiovasc Diabetol.

[9] Bamberg F, Parhofer KG, Lochner E, Marcus RP, Theisen D, Findeisen HM, Hoffmann U, Schönberg SO, Schlett CL, Reiser MF, Weckbach S (2013). Diabetes mellitus: long-term prognostic value of whole-body mr imaging for the occurrence of cardiac and cerebrovascular events. Radiology.

[10] Waugh SA, Ramkumar PG, Gandy SJ, Nicholas RS, Martin P, Belch JJF, Struthers AD, Houston JG (2009). Optimization of the contrast dose and injection rates in whole-body MR angiography at 3.0T. J Magn Reson Imaging.

[11] Nielsen YW, Eiberg JP, Logager VB, Schroeder TV, Just S, Thomsen HS (2009). Whole-body magnetic resonance angiography at 3 tesla using a hybrid protocol in patients with peripheral arterial disease. Cardiovasc Interv Radiol.

[12] Weir-McCall JR, Khan F, Lambert MA, Adamson CL, Gardner M, Gandy SJ, Ramkumar PG, Belch JJF, Struthers AD, Rauchhaus P, Morris AD, Houston JG (2014). Common carotid intima media thickness and ankle-brachial pressure index correlate with local but not global atheroma burden: a cross sectional study using whole body magnetic resonance angiography. PLoS One.

[13] Natori S, Lai S, Finn JP, Gomes AS, Hundley WG, Jerosch-Herold M, Pearson G, Sinha S, Arai A, Lima JAC, Bluemke DA (2006). Cardiovascular function in multi-ethnic study of atherosclerosis: normal values by age, sex, and ethnicity. AJR Am J Roentgenol.

[14] Mewton N, Opdahl A, Choi E-Y, Almeida ALC, Kawel N, Wu CO, Burke GL, Liu S, Liu K, Bluemke DA, Lima JAC (2013). Left ventricular global function index by magnetic resonance imaging–a novel marker for assessment of cardiac performance for the prediction of cardiovascular events: the multi-ethnic study of atherosclerosis. Hypertension.

[15] Burke AP, Kolodgie FD, Zieske A, Fowler DR, Weber DK, Varghese PJ, Farb A, Virmani R (2004). Morphologic findings of coronary atherosclerotic plaques in diabetics: a postmortem study. Arterioscler Thromb Vasc Biol.

[16] Moreno PR, Murcia AM, Palacios IF, Leon MN, Bernardi VH, Fuster V, Fallon JT (2000). Coronary composition and macrophage infiltration in atherectomy specimens from patients with diabetes mellitus. Circulation.

[17] Edsfeldt A, Gonçalves I, Grufman H, Nitulescu M, Dunér P, Bengtsson E, Mollet IG, Persson A, Nilsson M, Orho-Melander M, Melander O, Björkbacka H, Nilsson J. Impaired fibrous repair: a possible contributor to atherosclerotic plaque vulnerability in patients with type II diabetes. Arterioscler Thromb Vasc Biol. 2014;2143–50. 10.1161/ATVBAHA.114.303414 25035341

[18] Kubo T, Imanishi T, Takarada S, Kuroi A, Ueno S, Yamano T, Tanimoto T, Matsuo Y, Masho T, Kitabata H, Tsuda K, Tomobuchi Y, Akasaka T (2007). Assessment of culprit lesion morphology in acute myocardial infarction: ability of optical coherence tomography compared with intravascular ultrasound and coronary angioscopy. J Am Coll Cardiol.

[19] Spagnoli LG, Mauriello A, Sangiorgi G, Fratoni S, Bonanno E, Schwartz RS, Piepgras DG, Pistolese R, Ippoliti A, Holmes DR (2004). Extracranial thrombotically active carotid plaque as a risk factor for ischemic stroke. JAMA.

[20] Kawata T, Daimon M, Hasegawa R, Toyoda T, Sekine T, Himi T, Uchida D, Miyazaki S, Hirose K, Ichikawa R, Maruyama M, Suzuki H, Daida H (2013). Prognostic value of coronary flow reserve assessed by transthoracic Doppler echocardiography on long-term outcome in asymptomatic patients with type 2 diabetes without overt coronary artery disease. Cardiovasc Diabetol.

[21] Tuccillo B, Accadia M, Rumolo S, Iengo R, D’Andrea A, Granata G, Sacra C, Guarini P, Al-Kebsi M, De Michele M, Ascione L (2008). Factors predicting coronary flow reserve impairment in patients evaluated for chest pain: an ultrasound study. J Cardiovasc Med (Hagerstown).

[22] Weckbach S, Findeisen HM, Schoenberg SO, Kramer H, Stark R, Clevert DA, Reiser MF, Parhofer KG (2009). Systemic cardiovascular complications in patients with long-standing diabetes mellitus: comprehensive assessment with whole-body magnetic resonance imaging/magnetic resonance angiography. Invest Radiol.

[23] Hansen T, Ahlström H, Söderberg S, Hulthe J, Wikström J, Lind L, Johansson L (2009). Visceral adipose tissue, adiponectin levels and insulin resistance are related to atherosclerosis as assessed by whole-body magnetic resonance angiography in an elderly population. Atherosclerosis.

[24] Lehrke S, Egenlauf B, Steen H, Lossnitzer D, Korosoglou G, Merten C, Ivandic BT, Giannitsis E (2009). Katus H a: Prediction of coronary artery disease by a systemic atherosclerosis score index derived from whole-body MR angiography. J Cardiovasc Magn Reson.

[25] Pajunen P, Nieminen MS, Taskinen MR, Syvänne M (1997). Quantitative comparison of angiographic characteristics of coronary artery disease in patients with noninsulin-dependent diabetes mellitus compared with matched nondiabetic control subjects. Am J Cardiol.

[26] Syvänne M, Pajunen P, Kahri J, Lahdenperä S, Ehnholm C, Nieminen MS, Taskinen MR (2001). Determinants of the severity and extent of coronary artery disease in patients with type-2 diabetes and in nondiabetic subjects. Coron Artery Dis.

[27] Migrino RQ, Bowers M, Harmann L, Prost R, LaDisa JF (2011). Carotid plaque regression following 6-month statin therapy assessed by 3 T cardiovascular magnetic resonance: comparison with ultrasound intima media thickness. J Cardiovasc Magn Reson.

[28] Ito S, Suzuki T, Katoh O, Ojio S, Sato H, Ehara M, Ito T, Myoishi M, Kawase Y, Kurokawa R, Suzuki Y, Sato K, Toyama J, Fukutomi T, Itoh M (2004). The influence of diabetes mellitus on plaque volume and vessel size in patients undergoing percutaneous coronary intervention. Jpn Heart J.

[29] Maffei E, Seitun S, Nieman K, Martini C, Guaricci AI, Tedeschi C, Weustink AC, Mollet NR, Berti E, Grilli R, Messalli G, Cademartiri F (2011). Assessment of coronary artery disease and calcified coronary plaque burden by computed tomography in patients with and without diabetes mellitus. Eur Radiol.

[30] Hansen T, Ahlström H, Wikström J, Lind L, Johansson L (2008). A total atherosclerotic score for whole-body MRA and its relation to traditional cardiovascular risk factors. Eur Radiol.

[31] De Lorenzo A, Lima RS, Siqueira-Filho AG, Pantoja MR (2002). Prevalence and prognostic value of perfusion defects detected by stress technetium-99 m sestamibi myocardial perfusion single-photon emission computed tomography in asymptomatic patients with diabetes mellitus and no known coronary artery disease. Am J Cardiol.

[32] Langer A, Freeman MR, Josse RG, Steiner G, Armstrong PW (1991). Detection of silent myocardial ischemia in diabetes mellitus. Am J Cardiol.

[33] Burgess DC, Hunt D, Li L, Zannino D, Williamson E, Davis TME, Laakso M, Kesäniemi YA, Zhang J, Sy RW, Lehto S, Mann S, Keech AC (2010). Incidence and predictors of silent myocardial infarction in type 2 diabetes and the effect of fenofibrate: an analysis from the Fenofibrate Intervention and Event Lowering in Diabetes (FIELD) study. Eur Heart J.

[34] Lundberg C, Johansson L, Barbier CE, Lind L, Ahlström H, Hansen T (2013). Total atherosclerotic burden by whole body magnetic resonance angiography predicts major adverse cardiovascular events. Atherosclerosis.

[35] Sakuma H (2011). Coronary CT versus MR angiography: the role of MR. Radiology..

