# Supplementary material for: Cohort comparison study of cardiac disease and atherosclerotic burden in type 2 diabetic adults using whole body cardiovascular magnetic resonance imaging
Source: Cardiovasc Diabetol. 2015 Sep 18;14:122. doi: 10.1186/s12933-015-0284-2 (PMC4574534; doi:10.1186/s12933-015-0284-2)
Supplement: Supplementary file 1 — Additional file 1: Table S1. Breakdown of the inclusion criteria for each of the cardiovascular disease territories. [file 12933_2015_284_MOESM1_ESM.docx]

**Table S1: Breakdown of the inclusion criteria for each of the cardiovascular disease territories.**

|  | **Cardiovascular Inclusion Criteria** | **Description** |
| --- | --- | --- |
| **I** | **Coronary Artery Disease (CAD)** | - Non-fatal acute myocardial infarction - Hospitalised acute coronary syndrome - Resuscitated cardiac arrest, - Coronary artery bypass graft (CABG) - Coronary revascularisation procedure |
| **II** | **Cerebrovascular Disease** | - Non-fatal ischaemic stroke - Transient ischaemic attack (TIA) confirmed by specialist (excluding TIA not confirmed by specialist) - Haemorrhagic stroke, and stroke associated with a primary haematological disease e.g leukaemia, polycythaemia, blood disease, tumour, trauma, or surgical procedures were not included. |
| **III** | **Lower extremity arterial disease (LEAD)** | - Ankle-brachial index (ABI) < 0.9 with one or more of:   - Intermittent claudication   - Abnormal toe systolic pressure, pulse volume recordings, or transcutaneous oxygen measurements   - Vascular imaging demonstrating LEAD   - Prior lower limb vascular surgery (excluding venous procedures), angioplasty or above ankle amputation |
